# A novel *Methylomirabilota* methanotroph potentially couples methane oxidation to iodate reduction

**DOI:** 10.1002/mlf2.12033

**Published:** 2022-08-09

**Authors:** Baoli Zhu, Clemens Karwautz, Stefan Andrei, Andreas Klingl, Jakob Pernthaler, Tillmann Lueders

**Affiliations:** ^1^ Key Laboratory of Agro‐Ecological Processes in Subtropical Regions, Taoyuan Agroecosystem Research Station, Institute of Subtropical Agriculture Chinese Academy of Sciences Changsha China; ^2^ Chair of Ecological Microbiology, Bayreuth Center of Ecology and Environmental Research (BayCEER) University of Bayreuth Bayreuth Germany; ^3^ Department of Plant and Microbial Biology, Limnological Station University of Zurich Zurich Switzerland; ^4^ Department of Limnology and Bio‐Oceanography University of Vienna Vienna Austria; ^5^ Biocenter of the LMU Munich Plant Development & Electron Microscopy Planegg‐Martinsried Germany

## Abstract

Methane oxidizing microbes play a key role in reducing the emission of this potent greenhouse gas to the atmosphere. The known versatility of the recently discovered anaerobic *Methylomirabilota* methanotrophs is limited. Here, we report a novel uncultured *Methylomirabilis* species, *Candidatus Methylomirabilis iodofontis*, with the genetic potential of iodate respiration from biofilm in iodine‐rich cavern spring water. Star‐like cells resembling *Methylomirabilis oxyfera* were directly observed from the biofilm and a high‐quality metagenome‐assembled genome (MAG) of *Ca*. *M. iodofontis* was assembled. In addition to oxygenic denitrification and aerobic methane oxidation pathways, the *M. iodofontis* MAG also indicated its iodate‐reducing potential, a capability that would enable the bacterium to use iodate other than nitrite as an electron acceptor, a hitherto unrecognized metabolic potential of *Methylomirabilota* methanotrophs. The results advance the current understanding of the ecophysiology of anaerobic *Methylomirabilota* methanotrophs and may suggest an additional methane sink, especially in iodate‐rich ecosystems.

Methane oxidizing microbes are essential in controlling methane emissions from various environments. In addition to aerobic methanotrophs within the *Proteobacteria* and *Verrucomicrobiota*, anaerobic methantrophic archaea (the ANMEs) and bacteria within the *Methylomirabilota* (previously NC10 phylum), capable of anaerobic oxidation of methane (AOM), have been discovered during the last two decades. ANME archaea are suggested to oxidize methane via reverse methanogenesis^1^, using different electron acceptors, such as sulfate^2^, iron oxides^3^, nitrate and nitrite^4^, ^5^, with or without a syntrophic partner. In contrast, bacteria within the methanotrophic *Methylomirabilota* oxidize methane via a canonical methane monooxygenase‐dependent aerobic pathway, exclusively using nitrite as electron acceptor^6^, ^7^. *Methylomirabilota* methanotrophs are proposed to generate their own intracellular oxygen supply via nitric oxide (NO) dismutation into O_2_ and N_2_, catalyzed by a putative NO dismutase^8^. NO dismutase (*nod*) genes are widely distributed among diverse microbial lineages^9^. In addition to this peculiar metabolism, *Methylomirabilis oxyfera* was reported to display a characteristic polygonal cell shape in electron micrographs^10^. However, it remains to be shown whether other *Methylomirabilota* methanotrophs also show similar morphologies.

The diversity of *Methylomirabilota* methanotrophs as inferred from functional marker genes, such as particulate methane monooxygenase (*pmoA*)^11^ or *nod* genes^12^ seems limited, especially in comparison to the diversity of *Methylomirabilota* derived from 16S rRNA sequences^12^, ^13^. Hitherto, the dominant bacteria in denitrifying AOM cultures, for example^14^, ^15^, ^16^, as well as environmental microbes with supposed denitrifying methane‐oxidizing capability^17^, ^18^, were all closely related to *M. oxyfera*. Other environmental metagenome‐assembled genomes (MAGs) affiliated with the *Methylomirabilota* phylum did not indicate a denitrifying potential linked to methanotrophy^19^, ^20^. Recently, denitrifying AOM enrichment cultures containing *Methylomirabilota* bacteria were reported to reduce selenate^21^ or chlorate^22^ under methane oxidation. However, there was no direct evidence for the involvement of *Methylomirabilota* in these processes. Hence, our current understanding of the diversity and metabolic versatility of *Methylomirabilota* methanotrophs remains very limited.

Here, we report the MAG of a novel *Methylomirabilota* bacterium, *Candidatus Methylomirabilis iodofontis*, from methane‐oxidizing biofilms sampled under iodine‐rich mineral water in a subsurface spring cavern in Sulzbrunn, Germany. Iodine‐rich (>20 mg l^−1^) formation water from the subalpine Lower Marine Molasse enters the spring together with thermogenic methane, which accumulated up to 3000 ppm in the undisturbed microoxic cavern atmosphere^23^. Within the submersed biofilm at the cavern wall, transmission electron microscopy revealed peculiar star‐shaped microbial morphologies, resembling that of *M. oxyfera* (Figure 1A). In addition, 16S rRNA gene sequences related to that of *Methylomirabilis* spp. were retrieved via targeted PCR and cloning (Figure S1), consistent with previous results of 16S rRNA gene amplicon sequencing of the respective submersed biofilms, where reads of the *Methylomirabilota* (NC10) accounted for up to 10%^23^. These lines of evidence all indicate the presence of *Methylomirabilis* methanotrophs in the cave biofilm.

**Figure 1:**
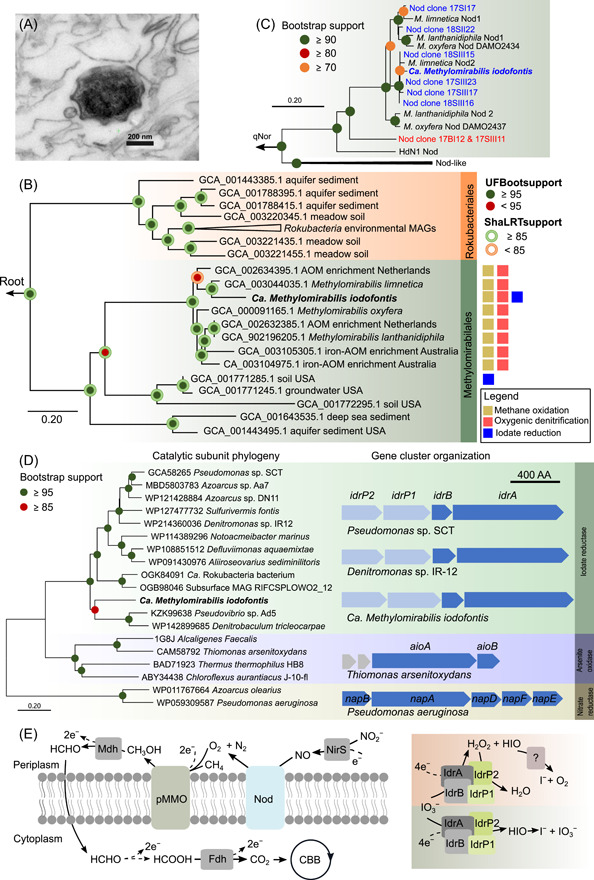
Cell morphology, phylogenetic analysis, gene cluster organization, and key respiratory pathways. (A) TEM image of *Methylomirabilis oxyfera*‐shaped cell from the submersed biofilm. (B) Phylogenomic analysis of *Methylomirabilota* phylum bacteria and MAGs, including *Methylomirabilis iodofontis* and other *Methylomirabilis* species and *Rocubacteriales*. (C) Nod phylogenetic tree including cloned Nod sequences from submersed biofilm and assembled Nod in *Candidatus Methylomirabilis iodofontis* genome. (D) Phylogenetic tree of the catalytic subunit of iodate reductase (IdrA), arsenite oxidase (AioA), and periplasmic nitrate reductase (NapA). IdrA encoded in the *M. iodofontis* is in bold, and the gene cluster organization of iodate reductase in *Pseudomonas* sp. SCT, *Denitromonas* sp. IR‐12 and *Ca*. *M. iodofontis*, and arsenite oxidase, nitrate reductase in other microbes are shown. (E) Key respiratory pathways in *M. iodofontis* according to genetic analysis. Both proposed iodate reduction routes taking place in periplasmic space are illustrated.

Therefore, we sequenced the metagenome of the submersed biofilm and assembled a putative *Methylomirabilota* genomic bin (bin48), which was over 70% complete and with very low contamination (1.52%) (Table S1). In total, 4780 *Methylomirabilota* 16S rRNA gene reads were retrieved, accounting for 14.3% of all 16S rRNA reads detected in the metagenomic library, representing one of the most abundant (sub)phylum‐level populations (Table S2). All *Methylomirabilota* 16S rRNA reads were assembled into one consensus full‐length 16S rRNA gene, which showed >99% similarity to that of *Methylomirabilis limnetica* (Figure S1). Yet, the pairwise average amino acid identity (AAI) and the average nucleotide identity (ANI) between *M. limnetica* genome and bin48 were only 85.8% and 91.3%, respectively, suggesting the newly binned MAG to represent a novel *Methylomirabilis* species, which was tentatively named *Ca*. *M. iodofontis*. Phylogenomic analysis based on 121 concatenated protein markers further supported that *M. iodofontis* was closely related to other *Methylomirabilis* species, forming a monophyletic clade within the order *Methylomirabilales* of the *Methylomirabilota* phylum (Figure 1B).

In the MAG of *M. iodofontis*, a pyrroloquinoline quinone (PQQ)‐dependent methanol‐dehydrogenase and a formate‐dehydrogenase highly similar to those in *M. oxyfera* and *M. limnetica* were also present. However, a particulate methane monooxygenase (pMMO) operon was missing (Table 1), possibly due to the incompleteness of the MAG. The presence of a complete methane‐oxidizing pathway in the MAG was statistically assessed using MetaPOAP^24^, and the false‐positive and false‐negative probabilities were 7.524e−10 and 0.069, respectively, suggesting that the pMMO genes are likely present in the source genome. Moreover, *M*. *iodofontis* harbored a complete Calvin−Benson−Bassham (CBB) cycle, except for the Rubisco small unit gene (Table 1), indicating an autotrophic lifestyle like *M. oxyfera* ^25^. The Rubisco large subunit of *M. iodofontis* clustered closely to that of other *Methylomirabilis* spp., all falling in the type IC/D group (Figure S2). The high similarity between *M. iodofontis* and other *Methylomirabilis* methanotrophs on the whole‐genome level as well as for key methane‐oxidizing enzyme genes (Table 1) also strongly argues for a methane‐oxidizing capability in *M. iodofontis*. Like other *Methylomirabilis* species, a complete oxygenic denitrification pathway was present, although a second *nod* (DAMO2434‐like) gene^12^ was not identified in the MAG (Table 1). Yet, *nod*‐targeted PCR and cloning recovered two Nod clusters as known for other *Methylomirabilis* spp., and a distantly related Nod (Figure 1C), indicating that the *M. iodofontis* genome likely also harbors two distinct *nod* gene homologs. The *M. iodofontis* Nod possessed all characteristic substitutions known for other Nod sequences (Figure S3). In comparison, reconstructed genomes of other members of the *Methylomirabilales* ^19^, ^20^, distantly related to *Methylomirabilis* spp., neither indicated methane oxidation nor oxygenic denitrification capacities (Figure 1B). Likely, the denitrifying methanotrophic lifestyle is restricted to the genus *Methylomirabilis* within the *Methylomirabilota*.

**Table 1:** Key metabolic pathways and CBB cycle‐associated genes in *Ca*. *Methylomirabilis iodofontis*.

Pathway	Locus tag	Gene	Enzyme	Similarity^a^ to gene of *M. oxyfera* (%)	Top hit (similarity)^a^
Oxygenic denitrification	bin‐48‐10‐cds15	*napB*	Nitrate reductase cytochrome c‐type subunit NapB	77.0	*M. limnetica* (83.5%)
	bin‐48‐10‐cds16	*napA*	Periplasmic nitrate reductase NapA	89.1	*M. limnetica* (92.5%)
	bin‐48‐132‐cds1	*nirS*	Nitrite reductase (NO‐forming)	88.5	*M. limnetica* (97.8%)
	bin‐48‐55‐cds1^b^	*nod*	Putative nitric oxide dismutase	83.8	*M. limnetica* (93.4%)
	bin‐48‐326‐cds1^b^	*nod*	Putative nitric oxide dismutase	91.0	*M. limnetica* (99.3%)
Methane oxidation	Missing	*pmoCAB*	Particulate methane monooxygenase		
	bin‐48‐119‐cds7	*mxaF*	Methanol dehydrogenase	96.4	*M. limnetica* (97%)
	Missing	*fea*	Formaldehyde activating enzyme		
	bin‐48‐146‐cds2	*fhcD*	Formylmethanofuran tetrahydromethanopterin formyltransferase	93.4	*M. oxyfera* (93.4%)
	bin‐48‐153‐cds5	*folD*	Methylene H_4_F dehydrogenase	89.6	*M. limnetica* (94.7%)
	bin‐48‐154‐cds2	*fdhA*	Formate dehydrogenase major subunit	87.9	*M. limnetica* (91.1%)
	bin‐48‐7‐cds14	*fdhD*	Formate dehydrogenase accessory protein	89.2	*M. limnetica* (93.3%)
CBB cycle	bin‐48‐50‐cds5	*rbcL*	Ribulose bisphosphate carboxylase, large chain, N‐terminal	87.5	*M. limnetica* (92.4%)
	bin‐48‐99‐cds1	*rbcL*	Ribulose‐bisphosphate carboxylase, large chain	96.9	*M. limnetica* (97.6%)
	Missing	*rbcS*	Ribulose‐bisphosphate carboxylase, small chain		
	bin‐48‐166‐cds1	*pgk*	Phosphoglycerate kinase	94.1	*M. oxyfera* (94.1%)
	bin‐48‐242‐cds3	*pgk*	Phosphoglycerate kinase	87.2	*M. limnetica* (95.2%)
	bin‐48‐108‐cds1	*gap*	Glyceraldehyde 3‐phosphate dehydrogenase	87.2	*M. limnetica* (96.6%)
	bin‐48‐166‐cds2	*gap*	Glyceraldehyde‐3‐phosphate dehydrogenase (NAD(P))	88.0	*M. limnetica* (98.8%)
	bin‐48‐242‐cds2	*tpi*	Triosephosphate isomerase	81.9	*M. limnetica* (93.4%)
	bin‐48‐242‐cds3	*tpi*	Triosephosphate isomerase	87.2	*M. limnetica* (95.2%)
	bin‐48‐99‐cds4	*fbb*	Fructose‐bisphosphate aldolase	ND	*M. limnetica* (95.6%)
	bin‐48‐99‐cds3	*fbp*	Fructose‐1,6‐bisphosphatase I	87.3	*M. limnetica* (94.4%)
	bin‐48‐108‐cds3	*glpX*	Fructose‐1,6‐bisphosphatase II	91.1	AOM enrichment (92.0%)
	bin‐48‐96‐cds4	*tkt*	Transketolase	ND	AOM enrichment (78.6)
	bin‐48‐108‐cds2	*tkt*	Transketolase	89.3	*M. limnetica* (95.5)
	bin‐48‐17‐cds6	*xfp*	Xylulose‐5‐phosphate/fructose‐6‐phosphate phosphoketolase	ND	AOM enrichment (85.0)
	bin‐48‐99‐cds2	*rpiA*	Ribose 5‐phosphate isomerase A	89.0	*M. limnetica* (91.8)
	bin‐48‐34‐cds1	*prk*	Phosphoribulokinase	94.8	*M. limnetica* (97.4)
Iodate reduction	bin‐48‐25‐cds2	*idrP2*	Cytochrome c peroxidase	34.1	Environmental MAG (58.5%)
	bin‐48‐25‐cds3	*idrP1*	Cytochrome c peroxidase	34.1	*Chloroflexi* bac. (61.1%)
	bin‐48‐25‐cds4	*idrB*	Arsenite oxidase small subunit	ND	*Rhodocyclaceae* bac. (55.7%)
	bin‐48‐25‐cds5	*idrA*	Arsenite oxidase large subunit	25.2	*Plancetomycetaceae* bac. (65.4%)

^a^ Based on amino acid sequence.

^b^ The two Nod sequences have 26 residual overlap and can be assembled, resulting in one complete *M. iodofontis* Nod. ND, no significant similarity found.

Interestingly, the cave spring water only contained low nitrate concentrations (<0.2 mg l^−1^) and nitrite was undetectable^23^. Thus, a potential for respiring other electron acceptors by *M. iodofontis* was assessed within the MAG. Remarkably, the corresponding MAG also harbored a gene cluster encoding cytochrome c peroxidases (IdrP1 and IdrP2) and an iodate reductase (IdrBA), the activity of which was recently demonstrated for *Pseudomonas* sp. SCT^26^ and *Denitromonas* sp. IR‐12^27^. The GC content and sequencing depth of the contig (bin48_25), where the iodate reductase gene cluster was located, was comparable to that of other contigs in the MAG, supporting its origin from *M. iodofontis* (Figure S4). Phylogenetic analysis demonstrated that the catalytic subunit of the iodate reductase (IdrA) of *M. iodofontis* was clearly placed within a cluster of iodate reductases (Figure 1D). The organization of this iodate reductase gene cluster (*idrP2,P1,B,A*) in *Ca. M. iodofontis* was also the same as in *Pseudomonas* sp. SCT and *Denitromons* sp. IR‐12 (Figure 1D). This organization seems characteristic among iodate reductases, distinct from more distantly related arsenite oxidases and periplasmic nitrate reductase encoding gene clusters^27^. These results strongly suggest that *M. iodofontis* carries a functional iodate reductase. Notably, *M. iodofontis* iodate reductase genes had no significant hits in genomes of other *Methylomirabilis* species (Table 1). An incomplete operon (*idrP1,B,A*) was detected on a contig of another subsurface *Methylomirabilota* MAG (GCA_001771285.1) (Figure 1B), which also belonged to the order *Methylomirabilales* but was not placed within the *Methylomirabilis* clade and lacked oxygenic denitrification and methane oxidation pathways (Figure 1B). This may indicate that *M. iodofontis* could have acquired iodate reductase genes via lateral gene transfer, as also proposed for other iodate‐reducing bacteria^27^.

SignalP analysis^28^ revealed that both IdrP1 and IdrP2 possess the Sec and IdrB possesses a twin‐arginine translocation (TAT) signal peptide, suggesting a periplasmic location of the *M. iodofontis* iodate reductase. This was also shown for *Pseudomonas* sp. SCT and *Denitromonas* sp. IR‐12^26^, ^27^. It has been proposed that in *Denitromonas* sp. IR‐12, IdrAB first reduces iodate to hypoiodous acid (HIO), which is chemically unstable and undergoes abiotic disproportionation to I^−^ and IO_3_^−^. The latter is subsequently cycled back to the enzymatic reduction^27^. In *Pseudomonas* sp. SCT, iodate reduction by IdrAB to hydrogen peroxide (H_2_O_2_) and HIO was proposed. The resulting H_2_O_2_ is detoxified by cytochrome c peroxidase (IdrP1 and IdrP2) to water and HIO is presumably disproportionated into O_2_ and iodide by a chlorite dismutase like (Cld‐like) enzyme^26^. Both *Denitromonas* sp. IR‐12 and *Pseudomonas* sp. SCT oxidize acetate to fuel iodate reduction; however, the potential electron donor for this reaction in *M. iodofontis* is still unclear. Notably, iodate reduction via the second proposed pathway would also allow for an oxygen‐dependent methane oxidation in *M. iodofontis* (Figure 1E), via the following redox reaction:

$$4{\mathrm{IO}}_{3}^{-}+3{\mathrm{CH}}_{4}\to 4I^{-}+6H_{2}O+3{\mathrm{CO}}_{2}$$

However, this metagenome‐derived physiology of *M. iodofontis* clearly awaits validation via labeling experiments in biofilm samples and enrichment cultures under laboratory conditions.

In summary, we report the MAG of a novel, yet uncultured *Methylomirabilota* methanotroph, *Ca*. *M. iodofontis*. Consistent with the specific biogeochemical setting of the iodine‐ and methane‐rich mineral spring cave, genetic and phylogenomic analyses suggest a capacity for methane oxidation, oxygenic denitrification, as well as iodate reduction in *M. iodofontis* (Figure 1E). This expands our perspective of the metabolic versatility of *Methylomirabilota* methanotrophs. Due to the ubiquity of iodate in ocean waters^29^, such ecophysiologies might be widely distributed and represent an overlooked methane sink in marine ecosystems.

## AUTHOR CONTRIBUTIONS

Clemens Karwautz, Baoli Zhu, and Tillmann Lueders obtained samples and did sequencing; Baoli Zhu, Stefan Andrei, and Clemens Karwautz analyzed the data; Andreas Klingl did EM; Baoli Zhu wrote the manuscript with inputs from Jakob Pernthaler and Tillmann Lueders. All authors read and approved the final manuscript.

## ETHICS STATEMENT

This study did not involve any human participant or animal subject.

## CONFLICT OF INTERESTS

The authors declare no conflict of interests.

## Supporting information

Supporting information.

## Data Availability

The metagenome sequences of this project were deposited at NCBI with accession number PRJNA825327.
