# Supplementary material for: A novel Methylomirabilota methanotroph potentially couples methane oxidation to iodate reduction
Source: mLife. 2022 Aug 9;1(3):323–8. doi: 10.1002/mlf2.12033 (PMC10989891; doi:10.1002/mlf2.12033)
Supplement: Supplementary file 1 — Supporting information. [file MLF2-1-323-s001.docx]

**Supporting Information**

Table S1. Overview of the *Ca*. Methylomirabilis iodoreducens MAG (bin48) information.

| **Size** | **GC (%)** | **completeness** | **contamination** | **NO. contigs** | **Total No. of genes** |
| --- | --- | --- | --- | --- | --- |
| 1.87M | ~60 | 73.8% | 1.52% | 330 | 1870 |

Table S2. Phyla with no less than three 16S reads in the metagenome.

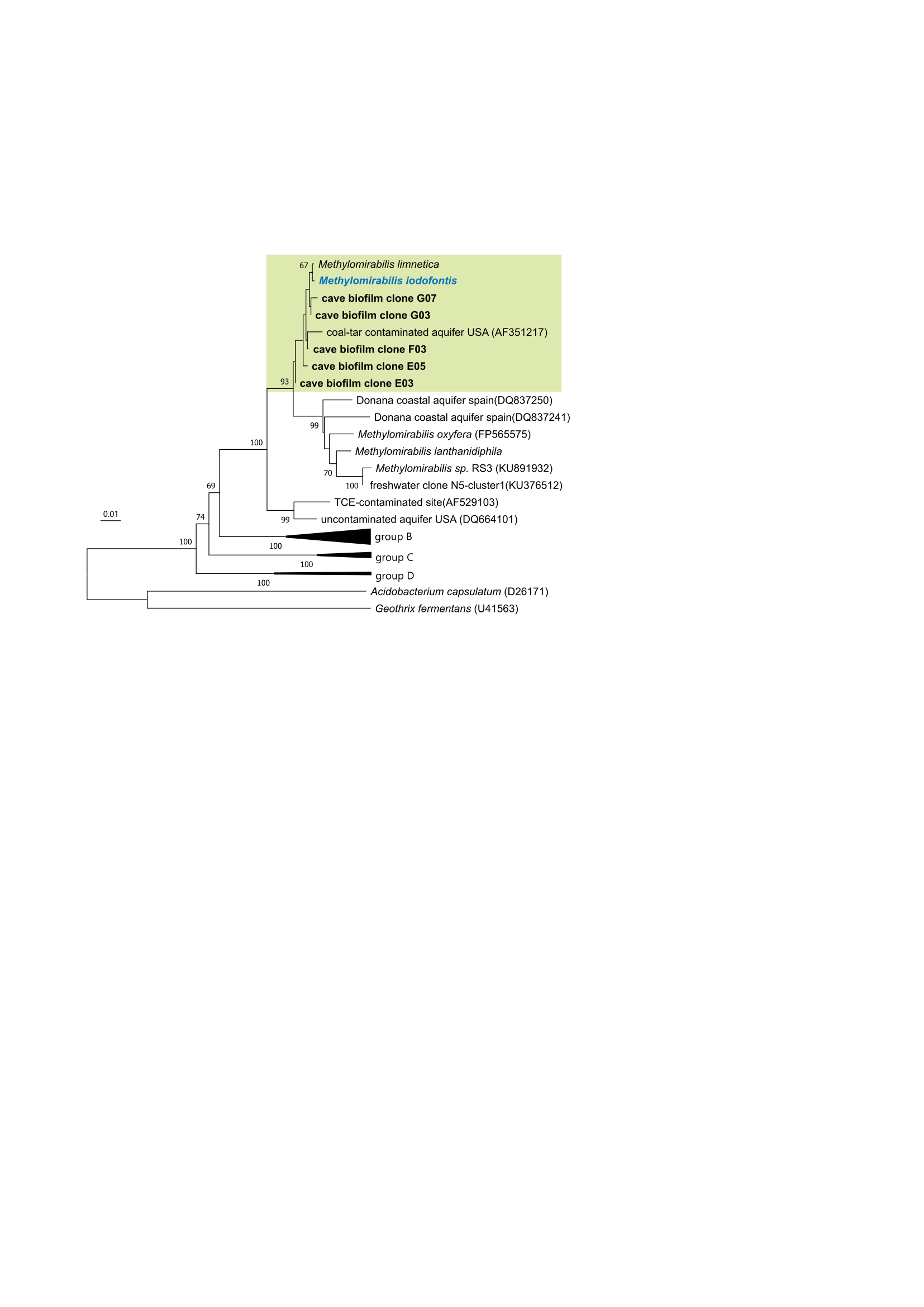


Figure S1. 16S rRNA phylogeny of *Methylomirabiliota* bacteria and environmental sequences. The assembled 16S rRNA of *Methylomirabilis* *iodofontis* is in blue, and biofilm clones in bold.

Figure S2 A maximum-likelihood phylogeny (LG+F+R10) of the large subunit of RuBisCo (types I-III) and RbuBisCo-like proteins (type IV) (n=147). Reference sequences were chosen based on previous tress constructed by Writghton et al. 2016 and Bulzu, Andrei et al. 2019 (n=146). The strength of support for internal nodes (assessed by Sh-aLRT test and ultrafast bootstrapping) is indicated through colored circles. Scale bar indicates the number of substitutions per site.


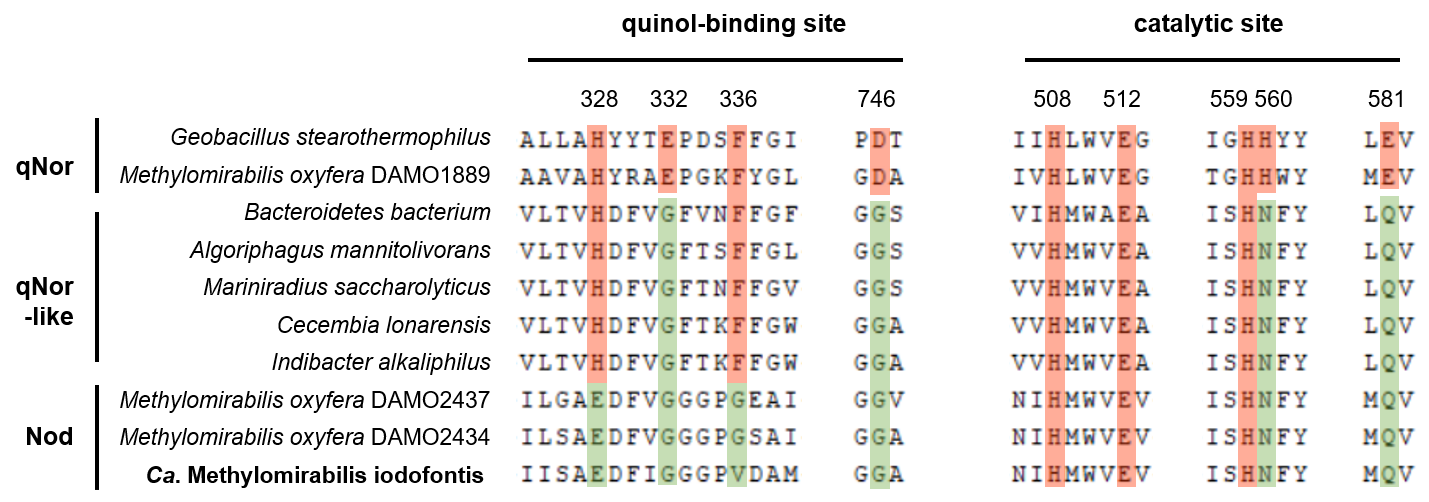


Figure S3. The Nod sequence of the *M. iodofontis* MAG possesses characteristic substitutions as other Nod sequences.

Figure S4. The GC vs coverage plot of all contigs from the *Ca*. Methylomirabilis iodofontis draft genome (bin48). The iodate reductase containing contig bin48_25 is shown in red.

## Methods

**Sampling**

Total DNA was extracted from biofilm samples as previously described (Karwautz *et al.*, 2018), metagenomic library construction and sequencing was conducted at the Josephine Bay Paul Center Marine Biological Laboratory (MBL), Woods Hole, MA, United States. In brief, Picogreen (ThermoFisher Scientific, Grand Island NY) was used to quantitate genomic DNA samples. 5-50 ng DNA was sheared using a Covaris S220 (Covaris, Woburn MA) and libraries were constructed according to the Ovation Ultralow Library v2 protocol (Nugen, San Carlos CA). Libraries were pooled at equimolar concentrations based on these results and size. The pool was quantified by qPCR (Kapa Biosystems, Wilmington, MA), and sequenced on an Illumina NextSeq platform. The assembly of the bin was done with MetaWrap which uses several assemblys.

**Transmission electron microscopy**

For transmission electron microscopy (TEM) the biofilms were fixed with 2.5% glutaraldehyde in 50 mM cacodylate buffer. Afterwards, the biofilms were postfixed with 1% osmium tetroxide for 80 min and dehydrated in a graded acetone series which included *en bloc* staining with 1% uranyl acetate in the 20% step. The cells were finally embedded in Spurr’s resin to enable ultrathin sectioning. To carry out electron microscopy, the ultra-thin sections were post-staining with 1% lead citrate for 2 min. The Zeiss EM 912 transmission electron microscope (Zeiss AG, Oberkochen, Germany) was set to an acceleration voltage of 80 kV and it was operated in the zero-loss mode. Images were acquired using a 2k x 2k slow-scan CCD camera (TRS Tröndle Restlichtverstärkersysteme, Moorenweis, Germany).

**Bioinformatic analysis**

Phylogenomics

The *Cand.* M. iodofontis MAG was screened for presence of collocated, lineage-specific marker sets by CheckM v1.0.11 (using the lineage_wf workflow) with the purpose of establishing its completeness and contamination. The obtained values (73.8 completeness and 1.52 contamination) rendered it suitable for taxonomic classification by Genome Taxonomy Database Toolkit (GTDB-Tk) v0.3.2 (default settings) with GTDB taxonomy (release 05-RS95). For more information regarding genome classification by relative evolutionary divergence and average nucleotide identity (or GTDB in general) see Chaumeil et al., 2019 and Parks et al., 2020. In order to obtain a broad view on the available genomic landscape of the Methylomirabilota phylum, we recovered (August 2020) publicly available genomes present in NCBI´s Microbial Genomes resource database (https://www.ncbi.nlm.nih.gov/genome/microbes/) and GTDB that were taxonomically classified as belonging to the phylum (n=40). The genomes belonging to this taxonomy-focused dataset (that comprises genomic data recovered from bacterial isolates, single-amplified genomes and environmental metagenomes) were used together with our obtained MAG to carry out evolutionary history inferences. Briefly, PRODIGAL v2.6.3 (default settings) was used to predict protein coding genes for each genome (n=44; 3 Bacteroidota genomes used as outgroup). The obtained proteomes were scanned with HMMER hmmscan v3.1b2 (with an E-value threshold set at 1e-5) against a locally installed TIGRFAMs (v15.0) database. A number of 121 ubiquitous single-copy protein sequences, that are usually employed in robust phylogenomic reconstructions (Parks *et al.*, 2020), were extracted from the genomes (based on TIGRFAMs accession numbers). These sets of unaligned homologous protein sequences were treated with PREQUAL v1.02 in order to mask possible non-homologous characters prior to alignment. Phylogeny-aware multiple sequence alignments (MSAs) were constructed for each phylogenomic marker (n=121) using the software PASTA v1.8.3 (Mirarab *et al.*, 2015) with default settings. The obtained alignments were trimmed with BMGE v1.12 (Criscuolo & Gribaldo, 2010), using the -g 0.5 setting, in order to retain aligned regions suitable for phylogenetic inferences. The 121 individual MSAs were concatenated in a supermatrix prior to performing genome-focused phylogenies. The phylogenomic tree was constructing using the maximum-likelihood algorithm implemented in IQ-TREE v1.6.10 (-m LG+C30+F+R10) by applying an LG amino-acid exchange rate matrix for 30 classes plus 31^th^ class of empirical AA profile (counted from the data) and a FreeRate heterogeneity model. The obtained tree was rooted with Bacteroidota phylum and visualised in FigTree v1.4.4 (available from <https://github.com/rambaut/figtree/releases>).

Phylogenetics

One protein belonging to the large subunit of the ribulose 1,5-bisphosphate carboxylase enzyme (RuBisCO) was identified in the assembled MAG. This protein, together with a dataset comprised of 146 RuBisCO (types I–III) and RuBisCO-like (type IV) proteins (Bulzu *et al.*, 2019) were treated with PREQUAL v1.02 prior to alignment with PASTA v1.8.3. The obtained alignment (854 aligned positions) was used to construct a maximum-likelihood phylogeny with IQ-TREE v.1.6.10 and the LG+F+R10 substitution model (chosen as the best-fitting model by ModelFinder).

16S rRNA abundance-based taxonomic classification

The obtained Illumina short reads were converted to FASTA format and queried, against the SILVA database (release 132, (Pruesse *et al.*, 2007)), in order to identify RNA-like sequences by using MMSeqs2 and an e-value cutoff of 1e-3. The bona fide 16S rRNA gene sequences (as identified by SSU-ALIGN) were further compared by blastn, in nucleotide space (using as cutoff the E-value 1e-5), against the SILVA SSU database (v138.1), and classified if the sequence identity was ≥80 % and the alignment length was ≥ 90 bp.

Genome annotation

Coding sequences for the recovered MAG were predicted de novo with PRODIGAL v2.6.3. BlastKOALA45 was used to assign KO identifiers (K numbers) to orthologous genes. Inferences of metabolic pathways and general biological functions were conducted with the online KEGG mapping tools (https://www.genome.jp/kegg/kegg1b.html) using summarized KO numbers assigned to each group. All predicted proteins were queried against NCBI NR, COGs (cluster of orthologous groups), TIGRs and Pfam databases. A locally installed version of InterProScan was used with default settings to annotate protein domains.

**References**

Bulzu P-A, Andrei A-Ş, Salcher MM, Mehrshad M, Inoue K, Kandori H, Beja O, Ghai R & Banciu HL (2019) Casting light on Asgardarchaeota metabolism in a sunlit microoxic niche. *Nat Microbiol* **4**: 1129-1137.

Criscuolo A & Gribaldo S (2010) BMGE (Block Mapping and Gathering with Entropy): a new software for selection of phylogenetic informative regions from multiple sequence alignments. *BMC Evolutionary Biology* **10**: 210.

Karwautz C, Kus G, Stockl M, Neu TR & Lueders T (2018) Microbial megacities fueled by methane oxidation in a mineral spring cave. *Isme Journal* **12**: 87-100.

Mirarab S, Nguyen N, Guo S, Wang L-S, Kim J & Warnow T (2015) PASTA: Ultra-Large Multiple Sequence Alignment for Nucleotide and Amino-Acid Sequences. *J Comput Biol* **22**: 377-386.

Parks DH, Chuvochina M, Chaumeil P-A, Rinke C, Mussig AJ & Hugenholtz P (2020) A complete domain-to-species taxonomy for Bacteria and Archaea. *Nature biotechnology* **38**: 1079-1086.

Pruesse E, Quast C, Knittel K, Fuchs BM, Ludwig W, Peplies J & Glöckner FO (2007) SILVA: a comprehensive online resource for quality checked and aligned ribosomal RNA sequence data compatible with ARB. *Nucleic acids research* **35**: 7188-7196.
